# Der Stellenwert der Thymektomie ohne Thymom in der Therapie der Myasthenia gravis

**DOI:** 10.1007/s00104-021-01436-3

**Published:** 2021-06-16

**Authors:** Hruy Menghesha, Michael Schroeter, Fabian Doerr, Georg Schlachtenberger, Matthias B. Heldwein, Costanza Chiapponi, Thorsten Wahlers, Christiane Bruns, Khosro Hekmat

**Affiliations:** 1 Klinik und Poliklinik für Herzchirurgie, herzchirurgische Intensivmedizin und Thoraxchirurgie, Universitätsklinik Köln, Universität zu Köln, Kerpener Straße 62, 50931 Köln, Deutschland; 2 Klinik und Poliklinik für Neurologie, Universitätsklinik Köln, Universität zu Köln, Köln, Deutschland; 3 Klinik und Poliklinik für Allgemein‑, Viszeral‑, Tumor-, und Transplantationschirurgie, Universitätsklinik Köln, Universität zu Köln, Köln, Deutschland

**Keywords:** Subgruppen, Acetylcholin-Rezeptor, Kausale Therapie, Minimal-invasive Therapie, Roboterassistierte Thymektomie, Subgroups, Acetylcholine receptor, Causal treatment, Minimally invasive treatment, Robotic-assisted thymectomy

## Abstract

Der Stellenwert der Thymektomie in der Therapie der thymomfreien Myasthenia gravis blieb bis vor einiger Zeit umstritten. Die relativ geringe Inzidenz und Prävalenz der Erkrankung, die uneinheitliche Dokumentation in den verschiedenen Studien sowie die notwendige Langzeitbeobachtung zur Erfassung therapeutischer Effekte erschwerten das Generieren valider Daten. Die Veröffentlichung des MGTX-Trials 2016 im *New England Journal of Medicine* lieferte die ersten randomisiert-kontrollierten Daten, nach denen Patienten mit Acetylcholin-Rezeptor-Antikörper-positiver generalisierter Myasthenia gravis im Alter von 18 bis 65 Jahren von der chirurgischen Resektion des Thymus über eine mediane Sternotomie profitieren. Trotz fehlender Validierung des Vorteils der Thymektomie über minimal-invasive Techniken durch randomisiert-kontrollierte Studien scheinen diese das Outcome bestimmter Patientengruppen in ähnlicher Form positiv zu beeinflussen. So haben videoassistiert-thorakoskopische, roboterassistierte, subxiphoidale und transzervikale Zugangswege nicht nur ästhetische Vorteile, sondern zeigen in der Beeinflussung des Krankheitsverlaufs der Myasthenia gravis keine relevante Unterlegenheit gegenüber der medianen Sternotomie. Doch nicht nur der Nutzen und das ästhetische Ergebnis differieren, sondern auch die Erfolgsaussichten im Hinblick auf die Remission sind bei den Unterformen der Myasthenia gravis unterschiedlich. Die heterogene Gruppe der Myasthenien unterscheidet sich bezüglich des Auftretens von Autoantikörpern, der betroffenen Körperregionen und des Alters der Patienten bei Erstdiagnose. Schließlich ist die Thymektomie eine wirksame kausale Therapie der Myasthenia gravis.

## Abstract (en)

The value of thymectomy in the treatment of non-thymomatous myasthenia gravis has been controversially discussed. The relatively low incidence and prevalence of this disease, the inconsistent documentation in various studies and the necessity of a long-term follow-up to assess the therapeutic effects has made the generation of valid data difficult. The publication in 2016 of the MGTX trial in the *New England Journal of Medicine* delivered the first randomized controlled data in which patients aged 18–65 years with generalized myasthenia gravis and positive for acetylcholine receptor antibodies showed a significant benefit after surgical resection of the thymus via median sternotomy. Despite a lack of validation of the advantages of thymectomy by minimally invasive surgery from randomized controlled studies, this technique seems to positively influence the outcome of certain patient groups in a similar way. Video-assisted thoracoscopic surgery (VATS) and robotic-assisted thoracic surgery (RATS) using subxyphoidal and transcervical access routes showed not only esthetic advantages but also showed no relevant inferiority in the influence on clinical outcomes of myasthenia gravis compared to median sternotomy; however, not only the benefits and the esthetic results show differences but also the advantages in the various subtypes of myasthenia gravis show divergent prospects of success with respect to remission. The clinical spectrum of myasthenia is heterogeneous with respect to the occurrence of antibodies, the body region affected and the age of the patient at first diagnosis. Ultimately, thymectomy is an effective causal treatment of myasthenia gravis.

## Hintergrund

Während der tomographische Nachweis eines Thymoms unzweifelhaft eine Operationsindikation darstellt, ist die Entfernung der Thymusdrüse aus immunmodulatorischer Indikation ein Sonderfall chirurgischer Indikationen. Hierbei wird die Entfernung der Thymusdrüse unabhängig von ihrer Größe und ihres Kontrastmittelverhaltens – sogar ohne Nachweisbarkeit des Thymus in der Schnittbildgebung – indiziert, um den langfristigen Verlauf der Autoimmunerkrankung Myasthenia gravis positiv zu beeinflussen. Dieser Eingriff ist damit vom Charakter her elektiv und prophylaktisch, seine langfristigen Erfolgsaussichten sind kurzfristig den Operationsrisiken im engeren Sinn als auch einem kurzfristigen Risiko einer Verschlechterung der Myasthenie gegenüberzustellen.

Pathophysiologisch ist dieser Therapieansatz darin begründet, dass die krankheitsverursachenden Antikörper gegen den Acetylcholin-Rezeptor durch eine Autoimmunreaktion gegen strukturähnliche Oberflächenantigene von speziellen Thymusgewebezellen gebildet werden (sog. molekulares Mimikry). Während pathophysiologisch die Mechanismen gut definiert sind, waren bis vor einigen Jahren die veröffentlichten klinischen Daten lediglich Beobachtungen und Studien von niedrigem Evidenzgrad, meist Fallserien mit retrospektivem Charakter. Um den Erfolg dieses chirurgischen Eingriffs auf den Krankheitsverlauf zu validieren, fehlten prospektive, randomisiert-kontrollierte Daten. Dies hat sich mit Veröffentlichung des MGTX-Trials 2016 [66] grundlegend geändert.

Die folgende Abhandlung erhebt den Anspruch, eine umfassende, allgemeine Übersicht über die aktuellen chirurgisch-therapeutischen Indikationen und Resektionsoptionen bei bestehender thymomfreier Myasthenia gravis zu vermitteln.

## Suchstrategie

Als Grundlage der Erstellung der allgemeinen Übersichtsarbeit wurde am 10.01.2021 eine Medline-Suche mithilfe der PubMed-Oberfläche durchgeführt. Alle aufgeführten wissenschaftlichen Artikel bis zum Jahre 2021 wurden berücksichtigt. Die Suchbegriffe „thymectomy“ [All Fields] AND „non-thymomatous myasthenia gravis“ [All Fields] OR „minimally invasive“ [All Fields] AND „thymectomy“ [All Fields] wurden verwendet, um eine umfassende Literaturrecherche zu präsentieren.

## Anatomie und Lokalisation des Thymus

**Figure 1:**
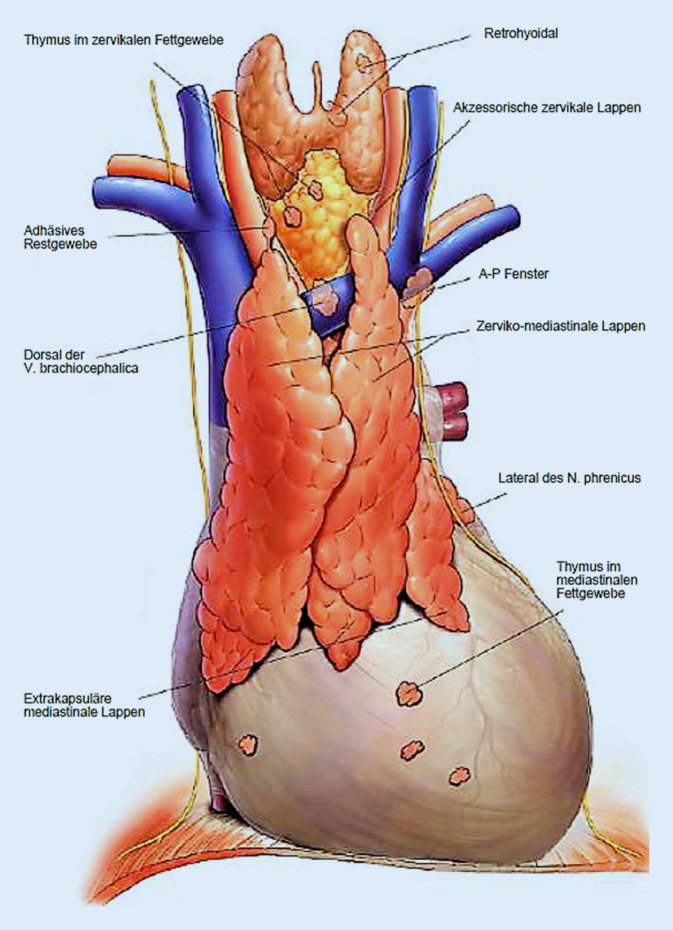

Der Thymus befindet sich im vorderen oberen Mediastinum. Die Begrenzungen werden apikal durch die Schilddrüse, kaudal durch das Diaphragma, beidseits lateral von den Nervi phrenici, ventral von dem Sternum und dorsal von den großen aszendierenden Gefäßen sowie dem venösen Konfluenz und der Vena cava superior begrenzt. Die Thymusdrüse besteht aus multiplen zervikalen und mediastinalen, häufig separaten Lappen. Zudem können sich winzige Foci im Bereich des prätrachealen und mediastinalen Fettgewebes verteilen (Abb. 1; [17, 45]).

## Die Anfänge der Thymektomie

Bereits Anfang des 20. Jahrhunderts wurde die Verbindung zwischen der Symptomatik einer Myasthenia gravis zu morphologischen Veränderungen des Thymus hergestellt [1, 21, 50]. Die erste Veröffentlichung einer therapeutischen Thymektomie im Rahmen der Behandlung einer Myasthenia gravis erfolgte bereits 1912 [57]. Publiziert wurde darin die Fallbeschreibung einer Patientin mit Hyperthyreose und Myasthenia gravis, die durch Ferdinand Sauerbruch einer Thyreoidektomie sowie einer Thymektomie unterzogen wurde und deren Symptome sich postoperativ regredient zeigten.

Blalock et al. beschrieben 1941 die positive Veränderung des Krankheitsverlaufs von Patienten mit Myasthenia gravis ohne Nachweis eines Thymoms nach vollständiger Resektion von Thymusgewebe [7].

## Nutzen der Thymektomie bei Myasthenia gravis

Im Jahr 2013 konnten Cea und Benatar et al. in ihrer Cochrane-Analyse die eingeschränkte Datenlage darstellen. Randomisierte prospektive Daten bezüglich der Thymektomie als Therapieoption bei Myasthenia gravis waren zu diesem Zeitpunkt nicht publiziert worden. Es entsprach jedoch bereits der gängigen Praxis, bei jungen Patienten mit einer generalisierten Acetylcholin-Rezeptor-Antikörper(AChR-Ak)-positiven Myasthenia gravis („early onset myasthenia gravis“, EOMG) eine Thymektomie vorzunehmen. Dabei wurden gute Effekte besonders dann beobachtet, wenn die Operation früh im Krankheitsverlauf, d. h. innerhalb der ersten 2 bis 5 Jahre nach Symptombeginn, durchgeführt wurde und die Patienten unter 40 Jahre waren. Der Pathologe findet in dieser Population dann überwiegend eine Thymushyperplasie mit Immunzellinfiltration des Organs als Zeichen des aktiven (auto-)immunogenen Prozesses.

Erst 2016 lieferte die Publikation des MGTX-Trials die erste Klasse-I-Evidenz für die Wirksamkeit der Thymektomie bei AChR-Ak-positiven Patienten [66]. Dabei wurde die Wirksamkeit der Behandlungsoption Thymektomie bei Patienten zwischen dem 18. und 65. Lebensjahr nachgewiesen und somit nicht nur bei EOMG-Patienten, sondern auch bei älteren, bei denen pathologisch meist lediglich eine Thymusinvolution zu finden ist („late onset myasthenia gravis“, LOMG). Die Wirksamkeit wurde gemessen als klinischer Vorteil (MGFA[Myasthenia gravis Foundation of America]-Score) und als Reduktion der notwendigen Prednisondosierung (kortisonsparender Effekt; [66]). Als Operationszugang wurde die mediane Sternotomie gewählt.

## Nutzen der Thymektomie bei AChR-Ak-positiven Patienten

Die MGTX-Studie hat klare Klasse-I-Evidenz für die Thymektomie bei AChR-Ak-positiver generalisierter Myasthenia gravis erbracht, was allerdings mögliche positive Effekte bei anderen Subformen der Myasthenia nicht ausschließt [20, 23, 34, 67].

In einem Leitlinienreport der American Academy of Neurology wird die Empfehlung zurückhaltend, jedoch zugunsten einer Thymektomie bei AChR-AK-positiven Patienten ausgesprochen [19]. Darüber hinaus sollte mit dem Patienten ein minimal-invasiver Zugang diskutiert werden.

Voraussetzung für die immunmodulatorische Wirkung ist die vollständige Entfernung des Thymusgewebes. Wird bei therapierefraktären Fällen eine Reoperation vorgenommen und Thymusgewebe gefunden und entfernt, so kann in Fallserien der klinische Verlauf positiv beeinflusst werden [37, 46].

Minimal-invasive Thymektomien bergen inhärent ein höheres Risiko als transsternale Operationen, dass Restthymusgewebe zurückbleibt. Bei therapierefraktären Fällen ist also insbesondere nach minimal-invasiven Voreingriffen eine transsternale Reoperation erwägenswert.

## Thymektomie bei anderen Patienten mit Myasthenia gravis

**Figure 2:**
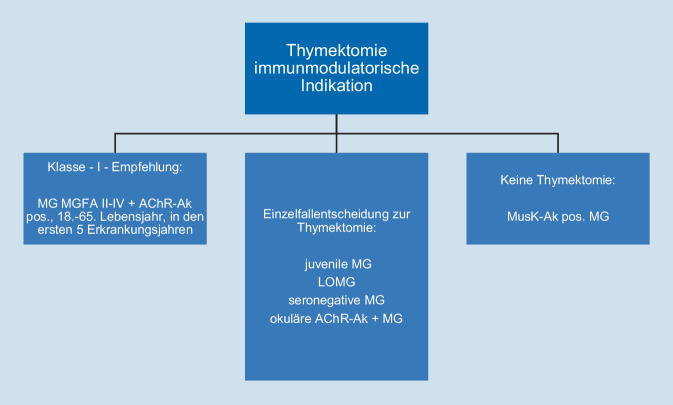

Etwa 1–5 % der Patienten mit generalisierter Myasthenie haben Antikörper gegen muskelspezifische Kinase (MusK; [18]). Bei dieser Subgruppe ist allgemein akzeptiert, dass die Durchführung einer Thymektomie nicht sinnvoll ist (Abb. 2; [8]). Neben sehr seltenen Fällen mit Nachweis von Lipoprotein-related-Protein-4(LRP4)-Antikörpern bleiben ca. 15 % der Patienten mit Myasthenie ohne Nachweis pathologischer Antikörper („seronegativ“). Vorherrschende Meinung ist, dass bei diesen Patienten durchaus Antikörper vorhanden sind, diese jedoch dem Nachweis im gängigen Labortest, der auf einer Kreuzaffinität der AChR-Ak gegen Bungarotoxinbindungsstellen beruht, entgehen [20, 67]. Yuan et al. beschreiben in ihrer Untersuchung, dass klinisch positive Effekte der Thymektomie zwischen seropositiven und seronegativen Patienten mit einer Myasthenia gravis ohne signifikanten Unterschiede sind [67].

Dennoch stellt die bislang einzige randomisierte kontrollierte Studie den Vorteil der Thymektomie gegenüber der Monotherapie mit Prednison nur bei seropositiven Patienten dar [66], was die Schlussfolgerung zulässt, dass eine Thymektomie auch bei seronegativen Patienten als Bestandteil eines individuellen Therapiekonzeptes erwogen werden sollte. Damit bleibt die chirurgische Resektion von Thymusgewebe bei seronegativer Myasthenia gravis eine Therapieoption für den Einzelfall.

## Nutzen bei Patienten mit okulärer Myasthenia gravis

Etwa 50 % aller neu diagnostizierten Myasthenien weisen eine reine okuläre Form auf [5, 54]. Das MGFA-Stadium I ist charakterisiert durch das Auftreten der krankheitstypischen Symptome einer Myasthenia gravis, wie Doppelbilder und Ptosis, welche durch Schwäche der extraokulären Muskulatur verursacht werden. Die Hälfte dieser Patienten zeigen eine Konversion zur generalisierten Form der Myasthenia gravis binnen 3 Jahre [4, 5]. Nur bei der Hälfte der Patienten mit okulärer Myasthenie lassen sich AChR-Ak nachweisen. Die aktuellen Therapieempfehlungen begründen sich ausschließlich auf die vorhandene retrospektive Datenlage [4, 47]. Dennoch scheint die Thymektomie die Generalisierungswahrscheinlichkeit zu reduzieren und damit einen signifikanten Einfluss auf die Entwicklung der Erkrankung dieser Patienten zu nehmen [36, 68]. Vor allem das Alter der Patienten bei Krankheitsbeginn, die dem operativen Eingriff unterzogen werden, und der Zeitraum von Krankheitsbeginn bis zur Thymektomie sind prognostisch hinweisgebend [26, 40, 47]. Junge Patienten mit okulärer Myasthenie und positivem AChR-Ak-Nachweis profitieren im frühen Krankheitsverlauf am wahrscheinlichsten von der Thymektomie [35]. Allerdings ist die Interpretation der veröffentlichten Daten nicht ohne Vorbehalt möglich, da das untersuchte Patientenkollektiv eine gewisse Inhomogenität in Bezug auf die analysierten Endpunkte und die Basischarakteristika aufweist [15], was wiederum in dem retrospektiven Charakter und der geringen Inzidenz und Prävalenz begründet ist.

## Thymektomie bei juveniler Myasthenia gravis

Die juvenile Form der Myasthenia gravis ist definiert durch einen Krankheitsbeginn vor dem 18. Lebensjahr [51]. Die Thymektomie wird äquivalent zur Therapie erwachsener Patienten mit thymomfreier Myasthenia gravis denjenigen empfohlen, die unter einer AChR-Ak-positiven generalisierten Form der Myasthenia gravis leiden [51]. Die durch Madenci et al. präsentierten Daten belegen gute Effekte der Thymektomie bei Patienten mit juveniler MG mit verhältnismäßig geringem Risiko einer postoperativen Komplikation [42].

## Thymektomie bei „late onset myasthenia gravis“

Obwohl klassisch die Thymektomie nur für EOMG-Patienten bis zum 40. Lebensjahr empfohlen wurde, zeigen die Ergebnisse des MGTX-Trials positive Effekte bis zum 65. Lebensjahr, also auch bei LOMG-Patienten [66]. Pathologische Ergebnisse zeigen zudem, dass die Altersgrenze zwischen EOMG und LOMG nicht strikt ist, sondern auch jüngere Patienten eine Thymusinvolution, eine LOMG definierend, aufweisen. Dagegen haben einzelne ältere Patienten eine Thymushyperplasie wie bei der EOMG [64]. Klinische Beobachtungen belegen, dass ältere Patienten ebenfalls von der Thymektomie profitieren, möglicherweise mit größerer Latenz als EOMG-Patienten. Auch retrospektive Daten unterstützen diese Ergebnisse, sowohl bei der Thymektomie via Sternotomie [62] als auch bei Durchführung einer roboterassistierten Thymusresektion [38].

## Operative Zugangswege

Die Debatte bezüglich des optimalen operativen Zugangs persistiert [41, 58, 59] nicht zuletzt aufgrund der Tatsache, dass sich die Thymusdrüse über zwei anatomische Regionen, der zervikalen und der mediastinalen, erstreckt [56].

Alle operativen Zugänge unterscheiden sich in Effektivität, kosmetischem Ergebnis, notwendiger Erfahrung des Operateurs und Kosten des Equipments [26]. Die chirurgische Komplexität besteht nicht zuletzt in der topographischen Variabilität des ektopen Thymusgewebes [2]. In der Regel besteht der Thymus nicht nur aus gut definierten, abgekapselten Lappen [7]. Das Thymusgewebe verteilt sich vielmehr zusätzlich diffus mediastinal und zervikal, was eine vollständige Resektion erschweren kann [25, 28].

**Figure 3:**
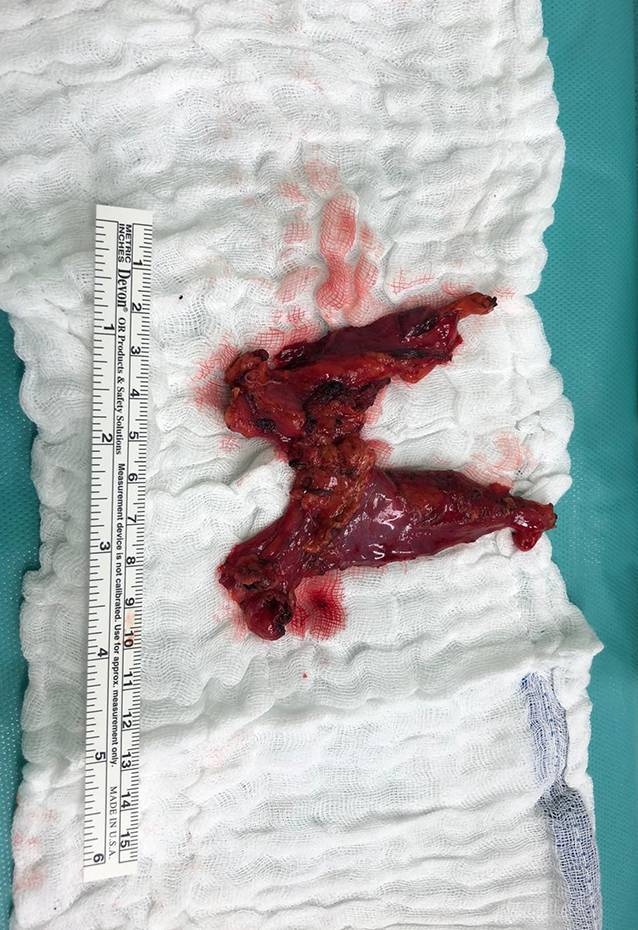

Die Möglichkeiten der vollständigen Resektion des Thymusgewebes sind unter den verschiedenen Operationszugängen nicht vergleichbar [25]. Das deklarierte Ziel besteht in der vollständigen Entfernung des Thymusgewebes (Abb. 3; [7, 10, 11, 14, 29, 31, 32, 53, 65]).

Demzufolge besteht der aktuelle Standard in der radikalen En-bloc-Resektion mediastinalen und zervikalen Thymusgewebes [28]. Darüber hinaus sollte parathymisches, mediastinales und perikardiales Fettgewebe entfernt werden, um, vor dem Hintergrund möglichen ektopen Thymusgewebes, das Outcome der Patienten hinsichtlich der Symptome einer bestehenden Myasthenia gravis zu verbessen [28, 39].

Die unvollständige Resektion der Thymusdrüse führte in diversen Untersuchungen zu einer Persistenz der Symptomatik und zur Notwendigkeit der Reoperation [22, 27, 33, 44, 46, 55]. Allerdings sind die Möglichkeiten, residuelles Thymusgewebe radiologisch darzustellen, limitiert, sodass die Indikationsstellung nicht selten klinisch erfolgt [49]. Referenzoperation bleibt die kombinierte transzervikale transsternale Thymektomie, welche durch Alfred Jaretzki in Anlehnung an den transsternalen Zugangsweg von Blalock konzipiert und propagiert wurde [28]. Dennoch wird das Interesse an minimal-invasiven Zugangswegen, welche mit signifikanten Vorteilen in Bezug auf den kosmetischen Aspekt, das Schmerzempfinden und konsekutiv den Analgetikagebrauch sowie den Hospitalisierungszeitraum einhergehen, stetig steigen.

Aufgrund der fehlenden intraoperativen Möglichkeit mikroskopisch kleine residuelle Thymusanteile darzustellen, ist die Gewissheit der Vollständigkeit einer Resektion nicht zu erlangen [25]. Die Qualitätskontrolle kann somit nur durch den Vergleich der kompletten stabilen Remissionsraten (CSR) erfolgen, wodurch die Erfolge der unterschiedlichen operativen Zugänge dargestellt werden können. Selbst minimale Restbestände von Thymusgewebe können eine Persistenz der Symptomatik bedingen [27].

Dennoch haben minimal-invasive Verfahren einen immer weiter wachsenden Stellenwert in der operativen Resektion von Thymusgewebe eingenommen.

Die anatomische Variabilität determiniert das Ausmaß der operativen Resektion. Die Thymusdrüse zeigt diverse Möglichkeiten der Ausbreitung im zervikalen und mediastinalen Bereich [45] bis hin zum subkarinalen Bereich auf [17].

### Transsternale Thymektomie

Die transsternale Thymektomie stellt eine in der ersten Hälfte des 20. Jahrhunderts initiierte Form der operativen Resektion des Thymusgewebes sowohl zur Therapie der thymomfreien Myasthenia gravis als auch den Behandlung der Thymustumoren dar [7]. Die Unterscheidung erfolgt weiterhin in eine Basisvariante sowie eine erweiterte Variante [48]. Nachdem die ausgeprägte Variabilität der möglichen Verteilung des Thymusgewebes dargestellt werden konnte, wurde dieses Verfahren der Thymektomie als einzige Möglichkeit der vollständigen Resektion antikörperbildender Foci angesehen [6, 7].

Der immer weiterwachsende Bedarf an Innovation und Minimalinvasivität machen dieses Operationsverfahren sowohl für den Behandler als auch den Patienten unattraktiver. Faktoren wie Ästhetik, Schmerztoleranz, Infektionsrisiko, Hospitalisierungsdauer und die Weiterentwicklung von Equipment bieten der Arzt-Patienten-Kommunikation diverse Auswahlmöglichkeiten mit unterschiedlich zugeschriebenen Eigenschaften der Resektionsvollständigkeit.

### Minimal-invasive Zugänge

Die minimal-invasive Thymektomie vereint folgende Operationszugänge [26]:

- den transzervikalen Zugang,
- den videoassistierten/roboterassistierten thorakoskopischen Zugang,
- den subxiphoidalen Zugang.

Die Videoskopie kann minimal-invasive zervikale, subxiphoidale und thorakoskopische Zugänge unterstützen und die Resektion von Thymusgewebe und mediastinalem Fettgewebe erleichtern [9, 12, 58, 69].

#### Transzervikale Thymektomie

Der zervikale Zugang, welcher als erste Form der Thymektomie im Jahre 1911 durch Sauerbruch an einer Patientin vollzogen wurde und der in den 1960er-Jahren von Kark et al. erneut als Standardzugang für die Thymektomie bei thymomfreien Myasthenia-gravis-Patienten deklariert wurde [30], zeichnet sich durch ein minimal-invasives Verfahren mit geringer ästhetischer Beeinträchtigung sowie geringer postoperativer Wundheilungsstörungstendenz aus [58]. Die Inzision erfolgt im Bereich des Jugulums. Durch Einsatz eines Retraktors ist die Konversion zu einem erweiterten Zugang möglich. Als alleiniges Operationsverfahren erscheinen beim klassischen transzervikalen Zugang eine suffiziente Visualisierung von Thymusgewebe und das damit einhergehende Ausmaß der Resektionsfähigkeit eingeschränkt. Das Risiko der Notwendigkeit einer Reoperation steigt damit signifikant [19].

### Subxiphoidale Thymektomie

Der infrastenale oder subxiphoidale Operationszugang ist sowohl singulär als auch in Kombination mit anderen Zugangswegen etabliert [24, 60, 63]. Die Ergebnisse der kombinierten transzervikalen subxiphoidalen Thymektomien erscheinen vergleichbar mit denen einer „maximalen“ transzervikalen transsternalen Thymektomie [69].

Der klare Vorteil in dem ausschließlich infrasternal gewählten Zugangsweg liegt in der Ästhetik. Allerdings ergeben sich aus dem engen Raum die fehlende Flexibilität der Manövrierbarkeit der Instrumente sowie die deutlich erschwerte Visualisierung, sodass er in den meisten Fällen nicht als Zugangsweg empfohlen wird.

#### Videoassistierte thorakoskopische Thymektomie

Die videoassistierte thorakoskopische Thymektomie (VATS-Thymektomie) kann sowohl als unilaterale als auch als bilaterale Version durchgeführt werden. Die signifikanten Vorteile gegenüber einer transsternalen Thymektomie liegen abgesehen von dem kosmetischen Aspekt in erster Linie im perioperativen Blutverlust, der Zeit, die die Patienten postoperativ auf einer Intensivstation verbringen müssen, der gesamten Hospitalisierungsdauer sowie der Operationsdauer [3], allerdings scheint die statistische Aussagekraft bezüglich des vergleichbaren Endpunktes der Remission der myasthenen Symptomatik nicht ausreichend zu sein [3, 19], sodass eine ausführliche Risiko-Nutzen-Abwägung mit dem zu behandelnden Patienten notwendig erscheint.

Tomelescu et al. und Manulu et al. präsentieren in ihren Abreiten valide Daten bezüglich der 10-Jahres-Remissionsraten von 75–88 % für die unilaterale VATS-Thymektomie [43, 61]. Auch die retrospektive Datenanalyse von Liu et al., in der die Remissionsrate von Patienten nach unilateraler VATS-Thymektomie mit der von Patienten, die mittels bilateralen Zugangs operiert wurden, verglichen wurde, suggerierte gleichwertige Ergebnisse [41], sodass eine eindeutige Überlegenheit eines minimal-invasiven Verfahrens in Bezug auf das Outcome bislang noch nicht mit ausreichender Evidenz dargestellt werden konnte [16].

#### Roboterassistierte Thymektomie

Seit Beginn des 21. Jahrhunderts wird die Thymektomie durch Unterstützung des Operationsroboters durchgeführt. Neben dem triportalen lateralen Zugang sind auch uniportale subxiphoidale Varianten etabliert. Auch bei diesem minimal-invasiven Zugang liegen die Vorteile gegenüber dem transsternalen Zugang in den ästhetischen Ergebnissen, dem geringeren Blutverlust bei ähnlicher Operationsdauer. Aufgrund der starken visuellen Vergrößerung sowie der ausgeprägten Flexibilität in der Ausrichtung der Operationsarme scheint die roboterassistierte Thymektomie der transsternalen Thymektomie im Hinblick auf die Radikalität in nichts nachzustehen und die der anderen videoassistierten Versionen sogar zu übersteigen [52]. Mittlerweile ist die roboterassistierte chirurgische Resektion nicht mehr ein experimentelles Verfahren, sondern ist im Begriff selbst für die Sonderformen der Myasthenia gravis als Mittel der Wahl anerkannt zu werden [36, 38].

Das Generieren randomisiert-kontrollierter Daten, um den Nutzen der roboterassistierten Thymektomie (RATS-Thymektomie) in direkten Vergleich zur Variante via Sternotomie zu setzen, erscheint äußerst unwahrscheinlich. Vor dem Hintergrund des immer weiterwachsenden Strebens nach Minimalinvasivität und Innovation wird der Anteil der Patienten, die sich sternotomieren lassen, während sie mittels Operationsroboter operiert werden könnten, verschwindend gering werden. Allerdings wäre ein Studiendesign ähnlich des MGTX-Trials denkbar, in dem die Sternotomie als Operationsverfahren durch die roboterassistierte Variante ersetzt werden könnte.

## Fazit für die Praxis

- Die Thymektomie ist fester Bestandteil der multimodalen Therapie der Myasthenia gravis.
- Trotz fehlender randomisierter Daten ist die Thymektomie in vielen Fällen als individuelles Therapiekonzept indiziert.
- Neben der medianen Sternotomie haben sich multiple operative Zugänge als mögliche Operationsverfahren durchgesetzt.
- Die roboterassistierte Thymektomie wird perspektivisch die Sternotomie als operativen Primärzugang vollständig verdrängen.
